# The computerized LASSI-BC Test versus the Standard LASSI-L Paper-and-Pencil Version in Community-Based-Samples

**DOI:** 10.4236/aad.2024.131002

**Published:** 2024-03-29

**Authors:** Rosie E. Curiel Cid, Alexandra Ortega, Ubbo Visser, Marcela Kitaigorodsky, D. Diane Zheng, Diana Hincapie, Kirsten Horne Crenshaw, Ashleigh Beaulieu, Brooke Bosworth, Liz Gallardo, Emory Neer, Sofia Ramirez, Elizabeth A. Crocco, Mike Georgiou, Efrosyni Sfakianaki, David A. Loewenstein

**Affiliations:** 1 Center for Cognitive Neuroscience and Aging and Department of Psychiatry and Behavioral Sciences, Miller School of Medicine, University of Miami, Miami, FL, USA; 2 Department of Computer Sciences, University of Miami, Coral Gables, FL, USA; 3 Nova Southeastern University, Fort Lauderdale, FL, USA; 4 Department of Radiology and Nuclear Medicine, Miller School of Medicine, University of Miami, Miami, FL, USA

**Keywords:** Mild Cognitive Impairment, Proactive Semantic Interference, LASSI-L, Computerized Cognitive Assessment

## Abstract

Proactive Semantic Interference (PSI) and failure to recover from PSI (frPSI), are novel constructs assessed by the LASSI-L. These measures are sensitive to cognitive changes in early Mild Cognitive Impairment (MCI) and preclinical AD determined by A*β* load using PET. The goal of this study was to compare a new computerized version of the LASSI-L (LASSI-Brief Computerized) to the standard paper-and-pencil version of the test. In this study, we examined 110 cognitively unimpaired (CU) older adults and 79 with amnestic MCI (aMCI) who were administered the paper-and-pencil form of the LASSI-L. Their performance was compared with 62 CU older adults and 52 aMCI participants examined using the LASSI-BC. After adjustment for covariates (degree of initial learning, sex, education, and language of evaluation) both the standard and computerized versions distinguished between aMCI and CU participants. The performance of CU and aMCI groups using either form was relatively commensurate. Importantly, an optimal combination of Cued B2 recall and Cued B1 intrusions on the LASSI-BC yielded an area under the ROC curve of .927, a sensitivity of 92.3% and specificity of 88.1%, relative to an area under the ROC curve of .815, a sensitivity of 72.5%, and a specificity of 79.1% obtained for the paper-and-pencil LASSI-L. Overall, the LASSI-BC was comparable, and in some ways, superior to the paper-and-pencil LASSI-L. Advantages of the LASSI-BC include a more standardized administration, suitability for remote assessment, and an automated scoring mechanism that can be verified by a built-in audio recording of responses.

## 1. Introduction

Alzheimer’s disease (AD) is a devastating condition that continues to significantly impact the rapidly aging population worldwide. It is widely acknowledged that novel emerging therapies will be most effective if applied before or during the earliest stages of AD-related Mild Cognitive Impairment (MCI) before multi-system brain degeneration has occurred [1] [2] [3]. While paper-and-pencil and computerized tests have been shown to be related to cognitive deficits in MCI, they have not been particularly sensitive to preclinical AD states [4] [5] [6]. Traditional measures have limited utility for this purpose because most have not shown specificity to A Dpathology such as amyloid-*β* (A*β*) and tau [7] [8], have not been widely validated with different ethnic/cultural groups [9], are vulnerable to practice effects, and often require a skilled examiner [5]. Further, statistically derived composite measures, while attempting to make optimal use of data collected from clinical trials, have been criticized for their reliance on cognitive paradigms developed in the 1940s and 1950s, do not provide a good theoretical rationale for weighting items, have not undergone rigorous reliability studies or exploration of their psychometric properties, may not be as equally beneficial for all ethnic and cultural groups and do not exploit the latest advances in cognitive neurosciences [10].

Our laboratory has been at the forefront of developing novel cognitive challenge tests or “cognitive stress” measures such as the Loewenstein-Acevedo Scales for Semantic Interference and Learning (LASSI-L) [11] [12] that are uniquely sensitive to the earliest AD-related changes in cognition in diverse cultural/language groups [9] and are highly related to specific brain biomarkers of AD (e.g., A*β*) [7] [8] [13]. From the standpoint of cognitive neuroscience, unique aspects of the LASSI-L, relative to existing measures, is a more robust measurement of proactive semantic interference (PSI) and most importantly, the failure to recover from PSI (frPSI). These subtle memory deficits among pre-symptomatic individuals on the AD continuum [5] [13] may be more sensitive than traditional list-learning measures [14].

Indeed, the LASSI-L differentiates between older adults who are cognitively unimpaired (CU) from those with clinically diagnosed early amnestic MCI (aMCI) and with Pre-MCI [5] [13] [15] much more effectively than other traditional neuropsychological measures and is also highly associated with brain A*β* load [7] [8], even among community-dwelling elderly with normal performance on widely used neuropsychological measures (preclinical AD) [14]. The LASSI-L is also specifically sensitive to atrophy in AD prone brain regions in older adults with aMCI [16] [17]. Importantly, Loewenstein *et al.* [8] found that semantic intrusions related to PSI and frPSI were considerably more pronounced in clinically diagnosed patients with aMCI with positive A*β* PET as opposed to aMCI patients with negative A*β* PET who met all other clinical criteria for AD. These results have been extended by Curiel Cid *et al* [18]. The LASSI-L has also been used internationally and has been superior to the commonly used Free and Cued Selective Reminding Test (FCSRT) in a Spanish population with early AD [14].

Additionally, frPSI semantic intrusion errors, but not other cognitive deficits, have been shown to be related to deficient limbic connectivity among asymptomatic middle-aged children of a parent diagnosed with AD in Buenos Aires, Argentina [17]. Recent work has led to the development of a computerized version of LASSI-L, the LASSI-BC [19]. The web-based LASSI-BC is reliable, valid and has the same discriminatory properties as the paper-and-pencil LASSI-L. The LASSI-BC does not require a skilled examiner, uses state-of-the-art voice recognition among English and Spanish-speaking older adults and because it is web-based, can be remotely accessed using computer or tablet technology.

In the current investigation, we examined two community-based cohorts of older patients who were diagnosed using identical clinical and neuropsychological criteria. Participants in one group were administered the paper-and-pencil LASSI-L while the other group received the computerized LASSI-BC. We hypothesized that both the LASSI-L and the LASSI-BC would be effective in differentiating those who were CU from those diagnosed with aMCI, and that both versions of the instrument would demonstrate PSI and frPSI effects, even when controlling for maximum learning capacity.

## 2. Methods

### 2.1. Participants

A total of 309 older adult participants from two NIA-funded R01 studies recruiting community-based older adults receiving an identical clinical and neuropsychological diagnostic battery were recruited into two IRB-approved investigations at the University of Miami Miller, School of Medicine. Participants had an average age of 71.88 years (SD = 8.1), with 64.98% being female. The average education level across all groups was 14.93 years (Range 5 - 21). Fifty-nine percent of participants were tested in English, while the remaining individuals were tested in Spanish, their dominant and preferred language for testing. All participants were evaluated using a standard clinical assessment protocol, which included the Clinical Dementia Rating Scale (CDR) [20], and the Mini-Mental State Examination (MMSE) [21]. Experienced clinicians who were blind to the neuropsychological test results and had formal training in administering the CDR to participants and a reliable informant to assess their memory and other clinical and cognitive complaints. All participants were community-dwellers, independent in their activities of daily living, had knowledgeable collateral informants, and did not meet DSM-V criteria for Major Neurocognitive Disorder, an active Mood or Psychotic Disorder, or any other neuropsychiatric disorder [22]. In cases where there was evidence of memory decline by history and/or clinical examination, the clinician scored the Global CDR as .5 and assigned a diagnosis of probable aMCI, pending the results of formal neuropsychological testing. Next, we administered a standard neuropsychological battery uniformly across groups independent of the clinical examination. Fluent Spanish/English bilingual psychometricians administered the test battery in the participants’ dominant and/or preferred language. Language preference for testing was determined by the participants’ self-assessment of language proficiency and preference. Literacy levels were assessed through standardized sight-word reading tasks. All Participants received either the standard paper-and-pencil LASSI-L or a computerized version, the LASSI-BC.

The neuropsychological battery used to classify older adults into diagnostic groups included widely-used standardized tasks of memory and other cognitive abilities and comprised the such as the MMSE, Hopkins Verbal Learning Test, revised (HVLT-R) [23] and delayed paragraph recall of the National Alzheimer’s Coordinating Center Uniform Data Set (NACC UDS) [24]. Controlled Oral Word Association Test: Category Fluency [25], Block Design subtest of the Wechsler Adult Intelligence Scale, Fourth Edition (WAIS-IV) [26], and the Trail Making Test (Parts A and B) [27].These tests were chosen for their documented sensitivity to cognitive changes among older adults at-risk for dementia, and their relevance to MCI research, ensuring a comprehensive and meaningful assessment. All tests underwent a double scoring process conducted by trained psychometricians to ensure the reliability of the scores. Clinical diagnoses were rendered through a multidisciplinary consensus conference that included a neuropsychologist and geriatric psychiatrist with a memory disorders expertise.

### 2.2. Amnestic MCI (aMCI) Groups

On the basis of the independent clinical interview and performance on the neuropsychological tests, an individual was classified as aMCI if there were: a) Subjective memory complaints by the participant and/or collateral informant; b) Evidence by clinical evaluation or history of memory and/or other cognitive decline; c) Global Clinical Dementia Rating scale of 0.5; d) One or more memory measures fell below normal limits at 1.5 SD or more relative to age, education, and language-adjusted normative data. Grouping for the Paper-and-pencil LASSI-L included a sample of 79 older adults with aMCI, while the LASSI-BC grouping included 52 individuals with aMCI.

### 2.3. Cognitively Unimpaired (CU) Groups

Participants were classified as cognitively unimpaired if all of the following criteria were met: a) No subjective cognitive complaints made by the participant and a collateral informant; b) No evidence by clinical evaluation or history of memory or other cognitive decline after an extensive interview with the participant and an informant; c) Global CDR score of 0; d) Performance on all traditional neuropsychological tests noted above was not more than 1.0 SD below normal limits for age, education, and language-adjusted normative data. Grouping for the paper-and-pencil LASSI-L included a sample of 110 older adults who were CU, while the LASSI-BC grouping included 67 individuals who were CU.

Table 1 depicts all demographic information for these four different groups. The mean age of the CU controls was slightly younger 71.1 (SD = 7.4 years) and these were slightly more educated than the aMCI cohort [15.4 (SD = 3.1 years)]. Female participants comprised the majority of 76.8% of this group. Sixty-seven percent were evaluated in English. The mean MMSE score was 26.00 (SD = 2.7).

### 2.4. Neuropsychological Measures

Neither the paper-and-pencil LASSI-L nor LASSI-BC were used for diagnostic determination in this study to avoid the circularity of confounding diagnosis with outcome. The LASSI-L has been extensively described previously [5] [7] [9] [11]. The LASSI-BC is the computerized version of the LASSI-L cognitive stress test, a novel cognitive assessment paradigm that employs controlled learning and cued recall to maximize the storage of a list of to-be-remembered target words representing three semantic categories [19]. This computerized measure, which is briefer than the paper-and-pencil LASSI-L, takes approximately 10 to 12 minutes to complete. The LASSI-BC contains the elements of the original LASSI-L which demonstrated the greatest differentiation between aMCI, PreMCI, and CU older adults in previous studies [13] [28]. The LASSI-BC is a remotely accessible test available in both English and Spanish. As a web application, it can be run on devices that can run Google Chrome, including desktop computers, laptops, tablets, or even smart phones. While the LASSI-BC is a fully self-administered test with all verbal responses recorded and scored by the computer, for the purposes of this study, a trained study team member was present for each administration to systematically record responses, which provided a double check on the accuracy of data. All psychometricians underwent training in the standardized administration and scoring of the instruments. While the LASSI-BC self-administers and scores, a trained psychometrist proctored the test and scored alongside the test to ensure that the data captured by the computer matched the data captured by a human. Importantly, all test administrations were then double-scored to ensurereliability. Notably, the computerized LASSI-BC version displayed robust test-retest reliability on key cognitive decline-sensitive subscales, effectively distinguishing aMCI from CU individuals during the initial validation studies. Significant correlations and optimal cut-points for aMCI discrimination, along with strong discriminatory power achieved by combining various LASSI-BC sub scores were suggestive that the LASSI-BC may be superior to the paper-and-pencil version. Logistic regression confirmed its high sensitivity (80%) and specificity (89.7%). The previous validation study concluded that the LASSI-BC surpassed the original LASSI-L, being feasible and exhibiting excellent discriminant properties. A thorough description of the LASSI-BC test and its psychometric properties can be found in Curiel Cid *et al.* [19].

### 2.5. Statistical Analysis

The distribution of demographic factors and neuropsychological measures were calculated and compared between the two diagnostics groups using the *χ*^2^ test for categorical variables and ANOVA for continuous variables. To examine aMCI and CU groups who received the LASSI-L versus LASSI-BC measures, the model was adjusted for statistically significant covariates such as sex, language of testing, and educational attainment. Controlling for demographic variables that initially differentiated the groups was an important step to adjust for variables that were measured but not randomized in the experiment. Subsequent analyses also adjusted for initial learning ability (A2 Cued Recall). Because initial learning also differentiated the groups, if we controlling for it ensure a more precise examination of the effects of proactive semantic interference (PSI) and the failure to recover from proactive semantic interference (frPSI). Logistic regression was performed to calculate sensitivity and specificity and examine the ability of both the paper-and-pencil LASSI-L as well as LASSI-BC measures of PSI and frPSI to differentiate CU from aMCI cases. The outcome variable of the logistic regression was the binary cognitive diagnosis (CU vs. aMCI). The area under the Receiver Operator Characteristic (ROC) curves were calculated for both LASSI-L and LASSI-BC.

## 3. Results

As indicated in Table 1, there were no statistical differences between diagnostic groups with regard to age. While CU participants did not differ with regard to their MMSE scores, aMCI participants who completed the computerized LASSI-BC had lower MMSE scores than their aMCI counterparts who completed the LASSI-L. There were group differences with regard to sex, the language of testing, and level of educational attainment (years of reported education). As mentioned in the Methods, these were entered as covariates in subsequent analyses to adjust for such effects.

As depicted in Table 2, in comparing different aMCI and cognitively unimpaired diagnostic groups on all LASSI measures, all p-values were <0.001. After adjusting for initial group differences in sex, educational attainment, and language of the evaluation, CU participants who completed the paper-and-pencil LASSI, had equivalent scores on Cued A2 Recall, Cued B1 Recall, Cued B2 Recall, and Cued A3 Recall and percentage of responses that were intrusion errors on Cued B1 Recall (PIE Cued B1) and Cued B2 Recall (PIE Cured B2) relative to participants who received the paper-and pencil-version. One exception was that CU participants made more Cued B1 Recall intrusions on the paper-and-pencil LASSI-L.

In contrast, aMCI individuals who received the computerized LASSI-BC, relative to those who received the paper-and-pencil LASSI-L evidenced less Cued A2 Recall (maximum learning), as well as lower Cued B2 Recall performance (failure to recover from proactive semantic interference) and percentage of intrusion errors (PIE) on Cued B2.In summary, all subtests of the computerized LASSI-BC differentiated between aMCI and CU participants while all subtests of the paper-and-pencil LASSI-L except Cued B1 Recall (measuring proactive semantic interference) differentiated aMCI from CU. Given the finding that Cued A2 Recall was lower for aMCI on the computerized LASSI-BC, Cued A2 Recall was entered into as a covariate in analyses to determine the effect on Cued B1 recall [proactive semantic interference (PSI)], Cued B2 Recall [ failure to recover from proactive semantic interference (frPSI)] and Cued A3 Recall (retroactive semantic interference). This was done to rule out the potential effects of initial learning on measures of PSI, frPSI, and RSI.

**Figure 1.**
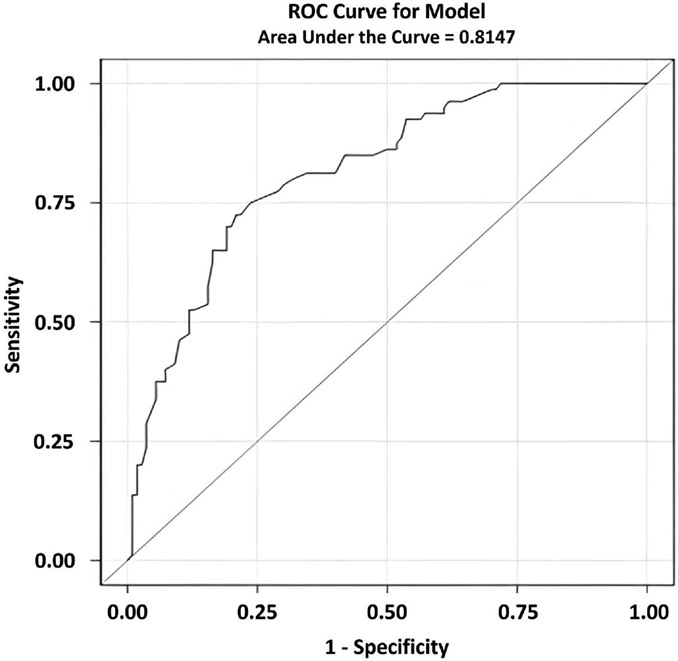
Area under the ROC curve for the paper-and-pencil LASSI-L.

As seen in Table 3, controlling for initial learning ability did not affect the ability of Cued B1 Recall to differentiate between aMCI and CU using either paper-and-pencil or computerized versions of the test demonstrating that initial learning did not account for the deficits observed on subtests that measured PSI and frPSI. Additionally, CU individuals evidenced equivalent performances on paper-and-pencil and computerized versions of the test. Conversely, a retroactive semantic interference effect (Cued A3) was no longer observed once initial learning was accounted for in the model. Logistic regression revealed that an optimal combination of Cued B2 recall and Cued B1 intrusions on the paper-and pencil LASSI-L produced a sensitivity of 72.5%, a specificity of 79.1%, and an area under the ROC curve of 0.815 (Figure 1).

**Figure 2.**
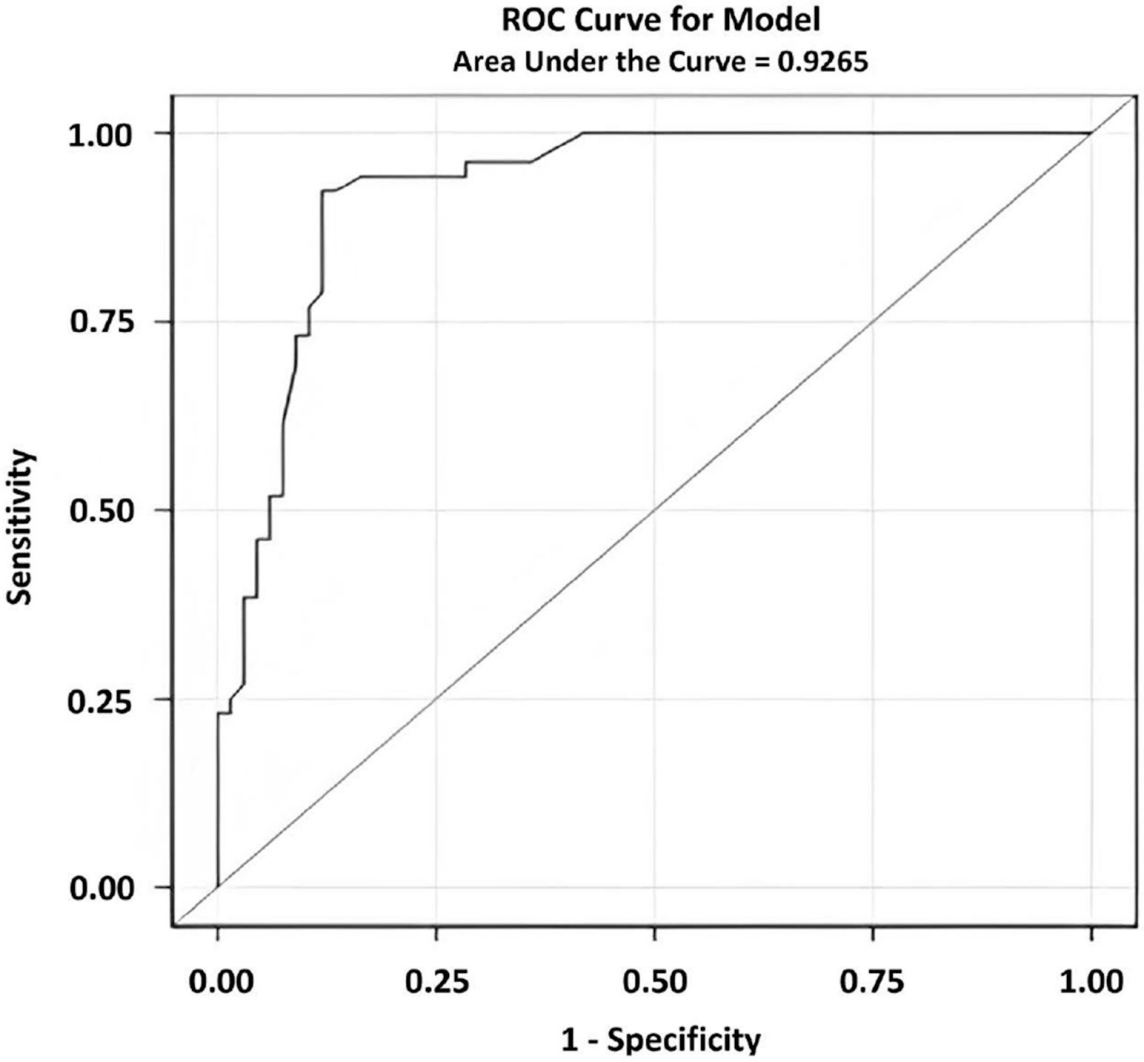
Area under the ROC curve for the computerized LASSI-L.

An optimal combination of Cued B2 recall and Cued B1 intrusions on the computerized LASSI-BC yielded a sensitivity of 92.3% and specificity of 88.1% and an area under the ROC curve of 0.927 for computerized LASSI (Figure 2). While area under the ROC curve were excellent for both versions of the LASSI-L and higher for the computerized LASSI-L, these could not be meaningfully compared given these measures were tested on two different samples which may have had different base-rates of underlying AD pathology.

## 4. Discussion

The failure to recover from proactive semantic interference on a paper-and-pencil version of the LASSI-L has successfully differentiated between older adults who are CU from those diagnosed with aMCI with underlying AD pathology based on FDG PET in Europe [14], and A*β* load on PET determined by visual reads and centiloid values in the U.S. [8] [13] [18]. This subtle cognitive deficit has even differentiated between middle-aged asymptomatic children of patients with late onset Alzheimer’s disease in South America and was related to cortico-limbic dysfunction in functional MRI (fMRI) [17]. Several studies have also found high correlations with structural MRI [16] and even combinations of biomarkers in path analytic models [29]. Previous investigators showed the LASSI-L could be completely computerized [19], had high test-retest reliabilities, and could significantly distinguish between aMCI and CU participants [9]. The advantages of computerizing this novel assessment paradigm are that the LASSI-BC enables improved standardization of administration, has automatic scoring capabilities (data capture using voice recognition software can be verified by a session recording), and has the ability for remote delivery by secure web-based platforms. The use of this technology to make this validated cognitive stress test paradigm widely accessible in both English and Spanish presents new opportunities for its implementation in AD clinical trials as it improves assessments using a paradigm that has evidenced robust sensitivity and high AD-specificity [9].

In the current investigation, we were able to comparatively examine two separate cohorts using the exact clinical and diagnostic criteria. Importantly, the two different LASSI-L paradigms were not used in diagnosis to avoid any potential circularity in our results. Furthermore, we adjusted several covariates for the purposes of statistically comparing participants who received the LASSI-L versus the LASSI-BC. There were two major issues that were addressed: 1) To determine the equivalency of the LASSI-L and LASSI-BC in CU and aMCI groups, 2) To ensure that both versions of the LASSI could distinguish between the aMCI and CU participants. We also conducted statistical adjustments to ensure that any effects captured on the LASSI’s unique measures tapping PSI and frPSI were not driven by initial learning ability.

Participants in either CU or aMCI groups could be differentiated after completing either the LASSI-L or LASSI-BC. This remained true after adjusting for demographic covariates, and even after adjusting for initial maximum learning on Cued A2 trials. This adjustment is important to ensure that all measures that are unique to the LASSI paradigm that measure susceptibility to PSI and frPSI would continue to have independent ability to distinguish between the aMCI and CU groups regardless of whether we employed the standard paper-and-pencil LASSI-L or the computerized LASSI-BC. The one exception was for intrusion errors on Cued B2 (measures PSI), which distinguished between CU and aMCI groups on the LASSI-L and LASSI-BC. However, adjustment for initial learning attenuated these findings for the LASSI-L.

For both the LASSI-L and LASSI-BC a combination of Cued B2 recall and Cued B1 intrusions yielded the optimal classification between diagnostics groups. However, the area under the ROC curve appeared to be superior for the LASSI-BC relative to the LASSI-L (0.927 versus 0.815). At first glance, one might argue that the LASSI-BC outperformed the LASSI-L with regards to differentiating CU and aMCI participants on Cued B2 intrusions. This may be an over-simplification in that two different cohorts were employed and that aMCI participants in each cohort may have had a different admixture of underlying etiologies for their aMCI. The LASSI-L number of intrusion errors has been shown to be extremely sensitive to degree of A*β* accumulation in the brain [18] with exceptionally high areas under the ROC curve in differentiating preclinical AD from normal controls. Lastly, it has also been shown that the LASSI-L measures have done an excellent job in identifying aMCI persons who progress to dementia and PreMCI persons who progress to aMCI, versus those do not progress or who revert to normal cognition over a two-to-three-year follow-up [28]. In the cohorts of aMCI participants examined in the current investigation, there were not enough participants with A*β* PET scans to determine potential admixtures of AD versus other pathologies. Follow-up PET scans on more participants can help to address these issues in future studies.

When comparing the paper-and-pencil versions of the LASSI-L versus the computerized version of the paradigm (LASSI-BC), we judged that since these involved the exact same learning stimuli, gathering two separate cohorts which received these measures was the most prudent way to evaluate the two important questions in the current study. However, it is acknowledged that the base-rates of underlying AD in the two community samples may bedifferent. Nonetheless, the present results indicate that both the LASSI-L and LASSI-BC could discriminate between CU and aMCI participants and that mean performance on the paper and pencil and computerized versions appeared to be relatively equivalent for CU and aMCI older adult participants. As we continue to develop alternate forms for both computerized and paper-and-pencil versions of the LASSI-L, we may ultimately be able to directly test paper-and-pencil forms and computerized versions of the instrument with the same individual over sufficient testing intervals.

The LASSI-L has been validated in different ethnic and cultural groups [9] [30] [31], has been related to a plethora of biomarkers in AD and has shown predictive validity over time [28]. The current results, while encouraging, suggest the need to conduct further extensive biomarker studies for the LASSI-BC. Given its ease of digital administration, and the ability to be delivered remotely through secure web-based platforms, we are optimistic that the paradigmatic similarity of LASSI-BC makes the measure a very promising candidate to move the field forward as it bridges an effective and novel cognitive assessment paradigm with the technological enhancements needed to optimize critically needed outcome measures.

## Figures and Tables

**Table 1.** Demographic Characteristics of amnestic Mild Cognitive Impairment (aMCI) and Cognitively Unimpaired (CU) Participants using the paper-and-pencil LASSI-L versus the Computerized LASSI-BC.

	Paper- and-Pencil LASSI-L CU (n = 110)	Computerized LASSI-BC CU (n = 67)	Paper- and-Pencil LASSI-L aMCI (n = 79)	Computerized LASSI-BC aMCI (n = 52)	F-Value or X^2^	P-Value
Age (Range: 59 - 92)	71.88 (SD = 8.1)	69.82 (SD = 5.2)	72.01 (SD = 7.9)	72.94 (SD = 8.0)	1.88	0.134
Female Sex	75.5%	79.4%	51.2%	53.8%	21.12	<0.001
Education (Range: 5 - 21)	14.76^a^ (SD = 3.2)	16.63^b^ (SD = 3.2)	14.18^a^ (SD = 3.1)	14.13^a^ (SD = 3.9)	8.90	<0.001
Language of testing (English or Spanish)						
English	68.2%	65.67%	57.5%	46.2%	8.39	0.032
MMSE (Range: 23 - 30)	29.12^a^ (SD = 0.12)	29.07^a^ (SD = 1.0)	27.11^c^ (SD = 2.90)	26.23^d^ (SD = 2.1)	61.02	<0.001

* Means for each measure with different alphabetic superscripts are statistically significant.

**Table 2.** Scores for Different Diagnostic Groups on the Paper-and-Pencil LASSI-L versus Computerized LASSI-BC.

	Paper- and-Pencil LASSI-L CU (n = 110)	Computerized LASSI-BC CU (n = 67)	Paper- and-Pencil LASSI-L aMCI (n = 79)	Computerized LASSI-BC aMCI (n = 52)	F-Value Adjusted for Sex, Education, and Language	P-Value
Cued A2 Recall (Maximum Learning)	13.29^a^ (SE = 0.164)	13.27^a^ (SE = 0.215)	11.04^b^ (SE = 0.195)	9.83^c^ (SE = 0.239)	177.51	<0.001
Cued B1 Recall	7.54^a^ (SE = 0.240)	8.01^a^ (SE = 0.315)	5.42^b^ (SE = 0.285)	4.69^b^ (SE = 0.351)	25.00	<0.001
Cued B2 Recall	11.30^a^ (SE = 0.220)	11.33^a^ (SE = 0.328)	8.83^c^ (SE = 0.261)	7.25^b^ (SE = 0.231)	46.09	<0.001
Cued A3 Recall	8.14^a^ (SE = 0.231)	8.06^a^ (SE = 0.302)	6.55^b^ (SE = 0.274)	7.68^b^ (SE = 0.337)	15.47	<0.001
Cued B1 Intrusions	3.01^b^ (SE = 0.237)	1.89^a^ (SE = 0.310)	4.96^c^ (SE = 0.282)	4.45^c^ (SE = 0.346)	20.17	<0.001
Cued B2 Intrusions	1.98^a^ (SE = 0.194)	1.42^a^ (SE = 0.254)	3.23^b^ (SE = 0.230)	3.64^b^ (SE = 0.283)	15.70	<0.001
Cued A3 Intrusions	3.56^a^ (SE = 0.275)	3.02^a^ (SE = 0.358)	4.93^b^ (SE = 0.330)	5.85^b^ (SE = 0.400)	11.75	<0.001
PIE Cued B1	0.266^a^ (SE = 0.019)	0.188^a^ (SE = 0.025)	0.456^b^ (SE = 0.023)	.480^b^ (SE = 0.028)	30.87	<0.001
PIE Cued B2	0.143^a^ (SE = 0.014)	0.111^a^ (SE = 0.018)	0.254^b^ (SE = 0.016)	0.324^c^ (SE = 0.020)	27.95	<0.001

* Means are adjusted for covariates in the model (sex, education, language of testing). Means for each measure with different alphabetic superscripts are statistically significant using the Bonferroni Correction.

**Table 3.** Scores for Different Diagnostic Groups on the Paper-and-Pencil versus Computerized LASSI-BC Adjusting for Initial Learning.

	Paper- and-Pencil LASSI-L CU (n = 110)	Computerized LASSI-BC CU (n = 67)	Paper- and-Pencil LASSI-L aMCI (n = 79)	Computerized LASSI-BC aMCI (n = 52)	F-Value Adjusted for Initial Learning (Cued A2 Recall), Education, and Language	P-Value
Cued B1 Recall	7.54^a^ (SE = 0.240)	8.01^a^ (SE = 0.315)	5.42^b^ (SE = 0.285)	4.69^b^ (SE = 0.351)	4.65	0.003
Cued B2 Recall	10.60^a^ (SE = 0.213)	10.69^a^ (SE = 0.270)	9.50^b^ (SE = 0.247)	8.60^b^ (SE = 0.330)	8.58	<0.001
Cued A3 Recall	7.68 (SE = 0.239)	7.54 (SE = 0.301)	6.98 (SE = 0.276)	6.63 (SE = 0.368)	1.863	0.136
Cued B1 Intrusions	3.32^b^ (SE = 0.250)	2.17^a^ (SE = 0.317)	4.65^d^ (SE = 0.291)	3.86^bc^ (SE = 0.388)	9.82	<0.001
Cued B2 Intrusions	2.24^ab^ (SE = 0.204)	1.68^a^ (SE = 0.259)	2.97^bc^ (SE = 0.937)	3.11^a^ (SE = 0.316)	4.63	0.004
Cued A3 Intrusions	3.80^ab^ (SE = 0.924)	3.36^a^ (SE = 0.370)	4.68^b^ (SE = 0.340)	5.28^b^ (SE = 0.453)	3.43	0.017

* Means are adjusted for covariates in the model (Cued A2 maximum learning, sex, education, language of testing). Means for each measure with different alphabetic superscripts are statistically significant using the Bonferroni Correction.
